# ParaEM: antigen-aware sequence-based paratope prediction with expectation–maximization

**DOI:** 10.1093/bioinformatics/btag739

**Published:** 2026-10-05

**Authors:** Taegyu Kim, Changyun Cho, Inuk Jung, Sun Kim

**Affiliations:** Interdisciplinary Program in Artificial Intelligence, Seoul National University, Seoul 08826, Republic of Korea; AIGENDRUG Co., Ltd, Seoul 08758, South Korea; School of Computer Science and Engineering, Kyungpook National University, Daegu 41566, Republic of Korea; Interdisciplinary Program in Artificial Intelligence, Seoul National University, Seoul 08826, Republic of Korea; AIGENDRUG Co., Ltd, Seoul 08758, South Korea; Interdisciplinary Program in Bioinformatics, Seoul National University, Seoul 08826, Republic of Korea; Department of Computer Science and Engineering, Seoul National University, Seoul 08826, Republic of Korea

## Abstract

**Motivation:**

Accurate identification of antibody paratopes—the residues contacting the antigen—is vital for understanding antibody–antigen recognition, antibody engineering, and vaccine design. However, structure-based predictors require antibody structural inputs, which are often obtained from experimentally determined antibody–antigen complexes in benchmark settings but are generally unavailable for new sequences. Conversely, sequence-based predictors scale efficiently but remain largely antigen-agnostic, limiting their use of pair-specific antigen context.

**Results:**

We introduce ParaEM, an antigen-aware sequence-based paratope predictor that operates directly on antibody and antigen sequences. ParaEM leverages pretrained ESM3 embeddings to encode both sequences, augmenting antibody residues with trainable, complementarity-determining regions (CDR)-informed embeddings to introduce a biologically informed inductive bias. It models interactions via a residue-level compatibility scorer that defines latent antigen-anchor distributions. During training, a generalized Expectation–Maximization (EM) framework alternates between estimating label-conditioned posterior responsibilities (E-step) and performing gradient-based parameter updates (M-step). Evaluated across three established benchmarks, ParaEM outperforms existing sequence-based baselines in AUC-PR and achieves performance competitive with structure-based predictors without requiring explicit three-dimensional structural inputs. Ablation studies show consistent improvements from generalized EM optimization and an additional gain from CDR-informed embeddings. Antibody-side and antigen-information controls further show that antibody sequence carries substantial predictive signal, while the matching antigen provides additional predictive information. Together, these results support ParaEM as a scalable framework for incorporating matching-antigen context into sequence-based paratope prediction.

**Availability and implementation:**

ParaEM is available at https://github.com/kimtaegyuu/ParaEM. An archival snapshot of the software corresponding to this study is available on Zenodo at DOI: 10.5281/zenodo.22157100.

## 1 Introduction

### 1.1 Motivation

Antibodies recognize and neutralize foreign molecules (antigens) through highly specific molecular interactions (Janeway *et al*. 2001, Burton and Hangartner 2016). The antibody paratope, i.e. the set of residues that physically contact the antigen, is typically enriched in the six complementarity-determining regions (CDRs), although framework residues can also contribute to binding. Accurate paratope identification is therefore important for understanding antigen–antibody interactions, guiding antibody engineering, and informing vaccine development (Almagro and Fransson 2008).

Structure-based predictors, such as PECAN (Paratope and Epitope prediction with graph Convolution Attention Network; Pittala and Bailey-Kellogg 2020), Paragraph (Chinery *et al*. 2023), MIPE (Multi-modal contrastive learning and Interaction informativeness estimation-based method for Paratope and Epitope prediction; Wang *et al*. 2024), and ParaSurf (Papadopoulos *et al*. 2025), leverage three-dimensional (3D) antibody coordinates and can achieve high accuracy. However, structural inputs remain a practical bottleneck: only a limited number of antibodies have high-quality experimental structures, and modeling hypervariable loops, especially CDR-H3, remains challenging even with state-of-the-art predictors such as AlphaFold-Multimer and IgFold (Evans *et al*. 2021, Ruffolo *et al*. 2023). Benchmark evaluations using experimentally determined bound antibody structures rely on structural information that is generally unavailable in prospective sequence-only applications, where antibody structures must instead be predicted. In contrast, sequence-based methods such as Parapred, ParaAntiProt, and the recent Paraplume scale efficiently and avoid explicit structural inputs (Liberis *et al*. 2018, Kalemati *et al*. 2024, Athènes *et al*. 2026), but are antigen-agnostic and therefore do not explicitly incorporate pair-specific antigen context. Since paratope formation depends on antibody–antigen compatibility, incorporating the matching antigen may provide information beyond antibody sequence alone.

To address this gap, we introduce ParaEM, a sequence-based paratope predictor that explicitly incorporates antigen context without requiring explicit 3D antibody or antigen structures as input.

#### 1.1.1 The paratope prediction problem

Given a matching antibody–antigen pair, our goal is to predict a residue-level paratope probability for each antibody position while using the antigen sequence as interaction context. Antigen-aware interface learning is challenging because experimentally resolved antibody–antigen complexes remain limited, antigen sequences vary substantially in length and composition, and positive interface residues are sparse. Extending this setting to simultaneous paratope–epitope inference introduces additional challenges because supervision must be modeled on both molecular partners and explicit cross-molecular modeling involves an n×m residue-pair space for an antibody of length *n* and an antigen of length *m*. We therefore focus here on antigen-aware antibody paratope prediction while leaving joint paratope–epitope inference as an important future extension.

### 1.2 Related work

#### 1.2.1 Structure-based methods

Structure-based paratope predictors derive signal from 3D geometry and molecular interaction patterns. PECAN (Pittala and Bailey-Kellogg 2020) models antibody–antigen interactions using graph convolution and attention over complex structures, whereas Paragraph (Chinery *et al*. 2023) represents antibodies as residue-level structural graphs without explicit antigen modeling. ParaSurf (Papadopoulos *et al*. 2025) utilizes molecular-surface patches with 3D convolutional architectures, while MIPE (Wang *et al*. 2024) jointly models paratope–epitope interactions through multimodal contrastive learning.

#### 1.2.2 Sequence-based methods

Sequence-based approaches avoid explicit structural dependence and, therefore, scale naturally to large repertoires. Parapred (Liberis *et al*. 2018) uses convolutional and recurrent networks over antibody sequence features, whereas ParaAntiProt (Kalemati *et al*. 2024) leverages pretrained protein language model embeddings for residue-level paratope prediction. More recently, Paraplume (Athènes *et al*. 2026) combines residue embeddings from multiple general-purpose and antibody-specific protein language models with a multilayer perceptron for paratope prediction. Paraplume operates directly on antibody sequences without explicit structural input and is antigen-agnostic.

### 1.3 Our approach

We propose ParaEM, a sequence-based framework that incorporates matching-antigen context through a latent antigen-anchor formulation optimized with Expectation–Maximization (EM) (Dempster *et al*. 1977). Antibody and antigen sequences are encoded with ESM3 (Hayes *et al*. 2025), and antibody representations are augmented with trainable CDR-informed embeddings. A residue-level compatibility scorer produces antibody–antigen scores, and generalized EM alternates posterior estimation over latent antigen anchors with parameter updates. At inference, ParaEM predicts antibody paratope probabilities from sequence alone; latent anchors are explanatory antigen-side variables rather than explicit contact predictions. We further evaluate a structure-based baseline using predicted antibody structures to examine performance when experimental structures are unavailable. As a complementary exploratory *post hoc* analysis, LLM-assisted UniProt annotation is used to examine variation in ParaEM performance across antigen subgroups.

## 2 Materials and methods

### 2.1 Data

We evaluated ParaEM on the standard splits used in prior paratope-prediction studies.

- **PECAN.** This benchmark contains 460 antibody–antigen complexes with paired heavy/light chains, protein antigens, and resolution better than 3 Å. CD-HIT clustering at 95% sequence identity was used in the original benchmark; we use its 205/103/152 train/validation/test split (Li and Godzik 2006, Pittala and Bailey-Kellogg 2020).
- **Paragraph expanded.** This SAbDab-derived set contains 1086 non-redundant antibody–antigen pairs; we use the original 60%/20%/20% train/validation/test split without additional filtering (Dunbar *et al*. 2014, Chinery *et al*. 2023).
- **MIPE.** This SAbDab-derived set contains 626 complexes. We reserve the original 10% independent test set and perform five-fold cross-validation on the remaining 90%; each fold-trained model is evaluated on the same independent test set (Wang *et al*. 2024).

Across datasets, sequences are parsed from PDB structures; paratope labels mark antibody residues with any heavy atom within 4.5 Å of an antigen heavy atom; and CDRs are assigned using IMGT numbering (Lefranc *et al*. 2009).

### 2.2 Method

An overview of the model architecture is depicted in Fig. 1. Although ParaEM includes latent antigen-anchor assignments, training supervision is provided only at the antibody paratope-label level, without explicit residue-level interaction or epitope labels.

**Figure 1:**
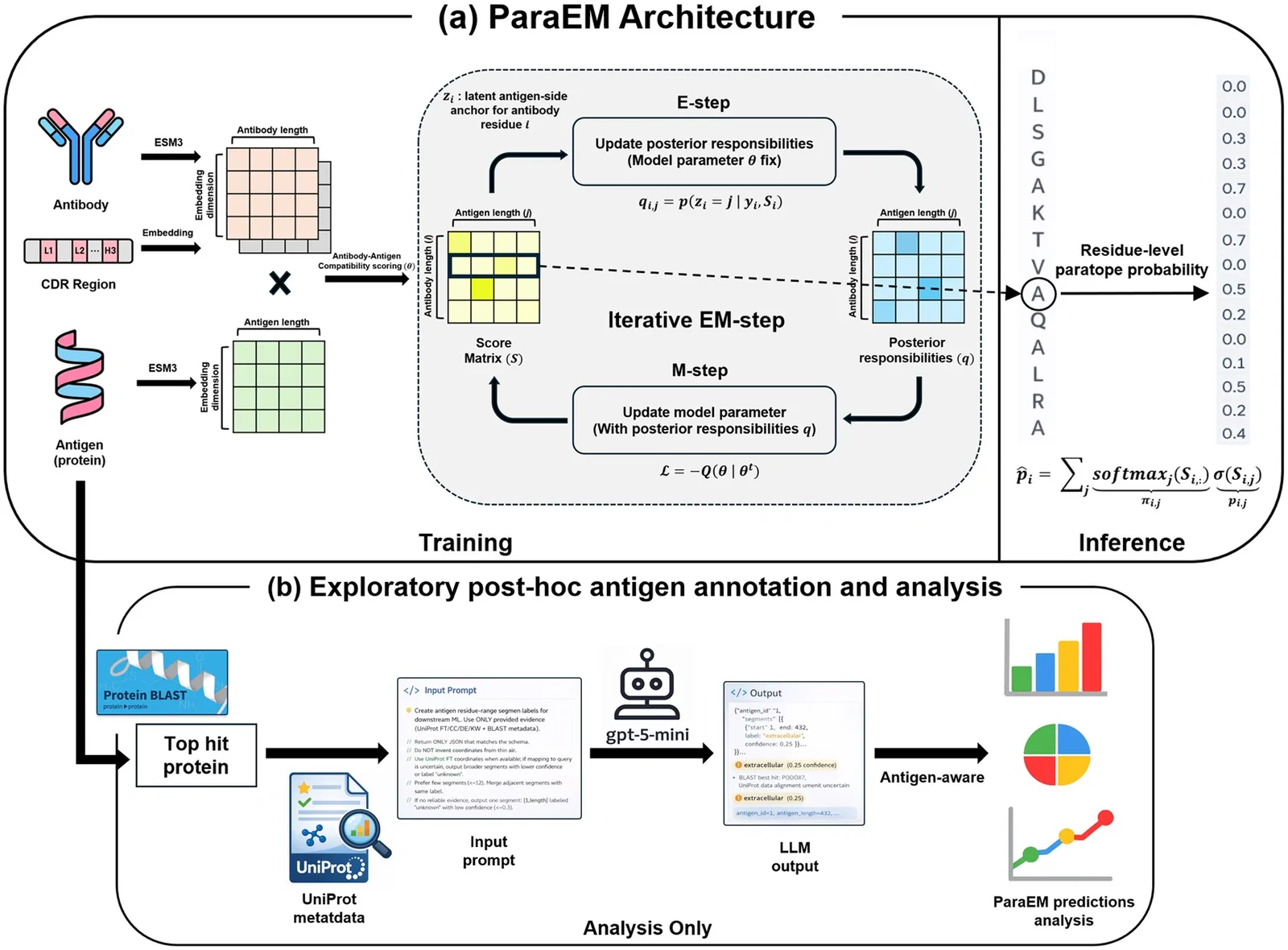
(a) Overview of ParaEM. Antibody and antigen sequences are encoded using a pretrained protein language model (ESM3), with antibody embeddings augmented by trainable CDR-informed embeddings. The compatibility scorer computes a residue–residue compatibility score matrix from antibody-derived queries and antigen-derived keys. During training, an EM procedure alternates between inferring posterior responsibilities over latent antigen anchors for each antibody position and updating model parameters with these responsibilities held fixed. At inference, row-wise aggregation of the compatibility score matrix yields per-residue paratope probabilities. (b) For exploratory *post hoc* analysis, each antigen is linked to UniProt via BLASTP. A protein-level LLM-assisted metadata annotation produces structured antigen descriptors used for subgroup stratification, while a separate residue-level annotation procedure is used only for topology-oriented diagnostics. Neither annotation procedure affects ParaEM training or inference.

#### 2.2.1 Embedding generation

Antibody and antigen sequences are encoded using the pretrained ESM3 protein language model, producing *D*-dimensional residue embeddings $e_{i}^{ab}\in R^{D}$ for antibody residues $i\in \{1,\ldots ,L_{\text{Ab}}\}$ and $e_{j}^{ag}\in R^{D}$ for antigen residues $j\in \{1,\ldots ,L_{\text{Ag}}\}$. For numerical stability, we apply learnable LayerNorm (Ba *et al*. 2016):


$${\tilde{e}}_{i}^{ab}=\text{LN}(e_{i}^{ab}),\;{\tilde{e}}_{j}^{ag}=\text{LN}(e_{j}^{ag}).$$

Each antibody residue *i* is assigned an IMGT category label $cdr_{i}\in \{0,\ldots ,6\}$, where 0 denotes a framework position and 1–3/4–6 denote the six CDR loops (H1–H3 and L1–L3). We represent this region identity with a trainable CDR-informed embedding using a lookup table $E_{\mathrm{cdr}}\in R^{7\times d_{\text{cdr}}}$:


$$c_{i}=E_{\mathrm{cdr}}[cdr_{i}]\in R^{d_{\text{cdr}}}.$$

#### 2.2.2 Antibody–antigen compatibility scoring

Given normalized antibody residue features ${\tilde{e}}_{i}^{ab}\in R^{D}$, antigen residue features ${\tilde{e}}_{j}^{ag}\in R^{D}$, and the CDR-informed embedding $c_{i}\in R^{d_{\text{cdr}}}$, we project antibody and antigen residues into a shared compatibility space of dimension $d_{\text{proj}}$:


$$\begin{array}{c} Q_{i}=W_{q}{\tilde{e}}_{i}^{ab}+W_{c}c_{i},\;W_{q}\in R^{d_{\text{proj}}\times D},\;W_{c}\in R^{d_{\text{proj}}\times d_{\text{cdr}}},\\ K_{j}=W_{k}{\tilde{e}}_{j}^{ag},\;W_{k}\in R^{d_{\text{proj}}\times D}. \end{array}$$

Dropout (Srivastava *et al*. 2014) is applied to Q and K during training. Pairwise antibody–antigen compatibility is computed as


$$S_{i,j}=\frac{\langle Q_{i},K_{j}\rangle }{\sqrt{Hd_{\text{proj}}}}.$$

In the implementation, Q and K are partitioned into *H* equal-sized blocks before averaging the scaled blockwise dot products, which is algebraically equivalent to the expression above. The resulting matrix $S\in R^{L_{\text{Ab}}\times L_{\text{Ag}}}$ contains residue–residue compatibility scores. A row-wise softmax of S defines a distribution over antigen positions for each antibody residue, which is used as the antigen-anchor distribution below.

#### 2.2.3 Latent antigen-anchor formulation and EM optimization

For each antibody residue *i*, we introduce a discrete latent antigen-anchor variable


$$z_{i}\in \{1,2,\ldots ,L_{\text{Ag}}\}.$$

$z_{i}$ indexes an unobserved antigen position over which the model marginalizes when predicting the antibody residue’s paratope state. It is an explanatory latent variable and is not supervised as a physical residue–residue contact. The observed paratope label is


$$y_{i}\in \{0,1\},$$

where $y_{i}=1$ indicates a paratope residue.

Given the antibody–antigen compatibility scores $S_{i,j}=S_{i,j}(\theta )$, we construct (i) an antigen-anchor distribution over antigen positions for each antibody residue,


$$\pi_{i,j}(\theta )={\text{softmax}}_{k}\left(S_{i,k}(\theta )\right),$$

and (ii) a Bernoulli evidence model for the observed label,


$$p\left(y_{i}|z_{i}=j,\theta \right)=\{\begin{array}{cc} \sigma \left(S_{i,j}(\theta )\right), & \text{if}\;y_{i}=1,\\ 1-\sigma \left(S_{i,j}(\theta )\right), & \text{if}\;y_{i}=0. \end{array}$$

##### 2.2.3.1 Prediction rule and direct BCE optimization

Because the antigen anchor $z_{i}$ is unobserved, we sum over all possible anchor positions (i.e. marginalize $z_{i}$) to obtain the residue-level paratope probability


$${\hat{p}}_{i}=p(y_{i}=1|\theta )=\sum_{j=1}^{L_{\text{Ag}}}\pi_{i,j}(\theta )\sigma (S_{i,j}(\theta )).$$

The non-EM control trains this same prediction rule directly using binary cross-entropy,


$$L_{\text{direct}}(\theta )=-\sum_{i=1}^{L_{\text{Ab}}}\left[y_{i}\;\text{log}\;\;{\hat{p}}_{i}+(1-y_{i})\;\text{log}(1-{\hat{p}}_{i})\right],$$

without an E-step or posterior responsibilities $q_{i,j}$. Thus, the Direct BCE and EM variants use the same compatibility scorer and the same prediction ${\hat{p}}_{i}$; they differ in how the model parameters are optimized.

##### 2.2.3.2 Complete-data likelihood

The joint model factorizes as


$$p(y_{i},z_{i}=j|\theta )=\pi_{i,j}(\theta )p(y_{i}|z_{i}=j,\theta ),$$

with complete-data log-likelihood


$$\text{log}\;p(y,z|\theta )=\sum_{i=1}^{L_{\text{Ab}}}\left[\text{log}\;\pi_{i,z_{i}}(\theta )+\text{log}\;p(y_{i}|z_{i},\theta )\right].$$

##### 2.2.3.3 E-step (posterior responsibilities)

With parameters $\theta^{\left(t\right)}$ fixed, we compute

1.
$\pi_{i,j}^{\left(t\right)}={\text{softmax}}_{k}(S_{i,k}^{\left(t\right)})$ ,
2.
$\ell_{i,j}^{\left(t\right)}=\sigma {\left(S_{i,j}^{\left(t\right)}\right)}^{y_{i}}{\left[1-\sigma (S_{i,j}^{\left(t\right)})\right]}^{1-y_{i}}$ ,
3. the label-conditioned posterior responsibility
  $$q_{i,j}^{\left(t\right)}=\frac{\pi_{i,j}^{\left(t\right)}\ell_{i,j}^{\left(t\right)}}{\sum_{k=1}^{L_{\text{Ag}}}\pi_{i,k}^{\left(t\right)}\ell_{i,k}^{\left(t\right)}}.$$

##### 2.2.3.4 M‐step (parameter update)

Holding the responsibilities $q^{\left(t\right)}$ fixed, we maximize the expected complete‐data log‐likelihood


$$\begin{array}{l} Q\left(\theta |\theta^{\left(t\right)}\right)=\sum_{i=1}^{L_{\text{Ab}}}\sum_{j=1}^{L_{\text{Ag}}}q_{i,j}^{\left(t\right)}[\text{log}\;\pi_{i,j}(\theta )\\ \;+y_{i}\;\text{log}\;\sigma (S_{i,j}(\theta ))+(1-y_{i})\;\text{log}\;\left(1-\sigma (S_{i,j}(\theta ))\right)]. \end{array}$$

Since a closed-form solution is unavailable for our model parameters θ, we perform gradient-based optimization on the negative objective


$$L_{\text{EM}}(\theta )=-Q\left(\theta |\theta^{\left(t\right)}\right),$$

as a generalized EM procedure. The E-step estimates label-conditioned posterior responsibilities $q^{\left(t\right)}$, which are held fixed during the subsequent gradient-based M-step updates. This separates latent antigen-side responsibility estimation from parameter updates within each EM iteration. Because supervision is available only for antibody-side paratope labels, different antigen-anchor assignments can yield similar marginal paratope probabilities; generalized EM provides a structured optimization procedure while leaving the predictive formulation unchanged.

##### 2.2.3.5 Inference

At test time, the same ${\hat{p}}_{i}$ is used as the residue-level paratope prediction, without the observed label $y_{i}$ or posterior responsibility $q_{i,j}$.

##### 2.2.3.6 Implementation details

For ESM3, the residue embedding dimension is D=1536, the IMGT vocabulary contains 7 categories, $d_{\text{cdr}}=32$, $d_{\text{proj}}=256$, H=4, and dropout is 0.1. We use frozen per-residue embeddings from ESM3 Open Small (esm3_sm_open_v1), precomputed offline without sequence truncation. Training follows 50 EM iterations; each iteration contains an E-step over all training pairs followed by two M-step optimization passes using per-complex updates (batch size 1). We use Adam (learning rate $10^{-5}$), with early stopping based on validation AUC-PR and report test performance from the validation-best checkpoint (Kingma and Ba 2014). Direct BCE uses the same architecture, random seed, hyperparameters, optimizer, schedule, and model-selection procedure without the E-step. Excluding ESM3 precomputation, training on one NVIDIA RTX A6000 typically completes within 10 minutes and inference on precomputed embeddings requires under 10 seconds per complex.

#### 2.2.4 Evaluation and analysis protocols

##### 2.2.4.1 Evaluation metrics and averaging

Paratope prediction performance is evaluated using the area under the precision–recall curve (AUC-PR) and the area under the receiver operating characteristic curve (AUC-ROC). Because paratope residues are sparse, AUC-PR is used as the primary evaluation metric. To avoid bias due to varying protein lengths, metrics are computed independently for each antibody–antigen complex and then averaged across complexes (macro-averaging).

##### 2.2.4.2 Contact-enrichment and anchor-localization diagnostics

To examine whether the learned compatibility score matrix *S* contains aggregate structure-related signal, we compare *S* with residue–residue contact maps derived from experimentally determined antibody–antigen complexes. We define $C_{i,j}=1$ when any heavy-atom distance between antibody residue *i* and antigen residue *j* is ≤4.5Å, and $C_{i,j}=0$ otherwise. Pair-level AUC-ROC measures whether $S_{i,j}$ tends to rank contacting residue pairs above non-contacting pairs, while the mean score difference


$$\Delta_{S}=E[S_{i,j}|C_{i,j}=1]-E[S_{i,j}|C_{i,j}=0]$$

quantifies the corresponding effect size.

Separately, we evaluate the localization of latent antigen anchors using Hit@*K*. For each complex, Hit@*K* is computed over antibody residues with at least one structural antigen contact and records whether any true contacting antigen residue appears among the top-*K* antigen positions ranked by the anchor weights $\pi_{i,j}$. We report macro Hit@*K*, obtained by averaging the per-complex Hit@*K* values across the test set. This diagnostic evaluates whether the latent anchors preferentially localize toward experimentally observed interface residues without interpreting them as explicit residue–pair contact predictions.

Both analyses are used only to characterize structure-related signal in the learned compatibility scores and latent anchors; neither residue–residue contact maps nor antigen-side epitope labels are used as training supervision.

##### 2.2.4.3 Antibody-side and antigen-information controls

We evaluated additional controls on PECAN to distinguish antibody-side predictive signal from antigen-specific context. First, a CDR-only heuristic assigned score 1 to residues annotated as IMGT CDRs and score 0 to framework residues, without model training. Second, an antibody-only ESM3 baseline used frozen antibody residue embeddings followed by layer normalization, a 256-dimensional projection matching the antibody-side projection in ParaEM, dropout, and a linear residue-level classifier trained with binary cross-entropy; antigen inputs and CDR-informed embeddings were omitted. Third, in the mean-antigen control, every antigen residue embedding in the training, validation, and test sets was replaced with the global mean of the training-set antigen embeddings, and ParaEM was retrained under the same configuration. Finally, in an antigen-pairing permutation control, test-set antigens were randomly reassigned across antibody–antigen complexes for 30 permutations while antibody sequences and residue-level labels were kept fixed. All controls were evaluated using the same macro-averaged AUC-PR and AUC-ROC implementation.

##### 2.2.4.4 Exploratory *post hoc* antigen annotation

For an exploratory *post hoc* analysis, each antigen sequence was linked to its top-ranked UniProt homolog (UniProt Consortium 2019) using BLASTP (Camacho *et al*. 2009). An LLM-assisted procedure organized the retrieved UniProt metadata into predefined descriptors, from which each antigen was assigned to one of four coarse antigen localization categories: viral surface, membrane, extracellular, or unknown. These annotations were used only for subgroup analysis and did not affect ParaEM training or inference. Annotation and residue-coordinate mapping details are provided in Text S2, available as supplementary data at *Bioinformatics* online; the deterministic UniProt cross-check is reported in Table S4, available as supplementary data at *Bioinformatics* online, subgroup resampling and permutation analyses in Table S3, available as supplementary data at *Bioinformatics* online, and the residue-level topology diagnostic in Text S4 and Fig. S1, available as supplementary data at *Bioinformatics* online.

## 3 Results

### 3.1 Performance on benchmark datasets

Table 1 summarizes performance across PECAN, Paragraph Expanded, and MIPE. ParaEM achieves consistently higher reported performance than existing sequence-based predictors across all three datasets. Among structure-based methods, only ParaSurf using experimentally determined bound antibody structures [ParaSurf (exp)] exceeds ParaEM on some benchmarks; these structural inputs are generally unavailable prospectively. Using a fixed probability threshold of 0.5 without dataset-specific tuning, ParaEM achieved macro F1/MCC values of 0.647/0.628, 0.684/0.662, and 0.626/0.611 on PECAN, Paragraph Expanded, and MIPE, respectively. Performance remained broadly comparable after excluding test antigens homologous to training antigens at 70%–40% identity thresholds (Table S1, available as supplementary data at *Bioinformatics* online).

**Table 1:** Paratope prediction performance (AUC-PR/AUC-ROC) on three benchmark datasets (PECAN/Paragraph Expanded/MIPE).^a^

Method	PECAN		Paragraph expanded		MIPE	
	AUC-PR	AUC-ROC	AUC-PR	AUC-ROC	AUC-PR	AUC-ROC
**Structure-based baselines (experimental structures)**						
PECAN	0.675	0.952	–	–	0.713	0.915
Paragraph	0.696	0.934	0.725	0.934	0.650	0.927
MIPE	–	–	–	–	0.741	0.927
ParaSurf (exp)	0.733	0.955	**0.793**	0.967	**0.781**	0.967
ParaSurf (pred)	0.715	0.948	0.723	0.957	0.717	0.956
**Sequence-based baselines**						
Parapred	0.646	0.930	–	–	0.652	0.868
ParaAntiProt	0.731	0.908	–	–	–	–
Paraplume	0.730	0.963	0.758	0.966	0.716	0.962
**ParaEM (Our method)**	**0.760**	**0.968**	0.786	**0.971**	0.757	**0.968**

a Sequence-based baselines use sequence inputs; structure-based baselines use experimental antibody structures. Except for ParaSurf (pred), external values are from the cited publications. Paraplume was independently reproduced, while its published values are retained in the table. ParaSurf (pred) uses ColabFold-predicted antibody structures. “–” indicates an unreported metric. Best values per metric are highlighted in bold.

### 3.2 Impact of predicted antibody structures on structure-based prediction

Replacing experimental with ColabFold-predicted (Mirdita *et al*. 2022) antibody structures reduced ParaSurf AUC-PR/AUC-ROC from 0.733/0.955 to 0.715/0.948 on PECAN, 0.793/0.967 to 0.723/0.957 on Paragraph Expanded, and 0.781/0.967 to 0.717/0.956 on MIPE. The larger AUC-PR reductions on Paragraph Expanded and MIPE indicate sensitivity to the antibody structure used as input. ParaEM avoids this structure-prediction step and instead operates directly on sequence. ColabFold details are provided in Text S1, available as supplementary data at *Bioinformatics* online.

### 3.3 Effect of EM optimization and CDR-informed embeddings

EM optimization improved AUC-PR over Direct BCE for both ESM2 (0.695–0.719) and ESM3 (0.716–0.748), with CDR-informed embeddings further increasing performance to 0.727 and 0.760, respectively (Table 2). For ESM3, 10 000 paired complex-level bootstrap resamples supported the EM gain (+0.032 AUC-PR, 95% CI [0.025, 0.036]; +0.021 AUC-ROC, [0.013, 0.028]) and the additional CDR-informed gain (+0.012 AUC-PR, [0.010, 0.013]; +0.005 AUC-ROC, [0.002, 0.008]).

**Table 2:** Effect of EM optimization and CDR-informed embeddings under different protein representations on the PECAN dataset.^a^

Method	ESM2		ESM3	
	AUC-PR	AUC-ROC	AUC-PR	AUC-ROC
Direct BCE	0.695	0.956	0.716	0.942
Direct BCE + CDR-informed embedding	0.701	0.957	0.723	0.949
EM	0.719	0.960	0.748	0.963
EM + CDR-informed embedding	**0.727**	**0.963**	**0.760**	**0.968**

a The best performance within each embedding representation is highlighted in bold. All variants use the same compatibility scorer and prediction rule.

### 3.4 Contribution of antibody-side and antigen information

The antibody-only ESM3 baseline substantially outperformed the CDR-only heuristic (AUC-PR 0.709 versus 0.326; Table 3), showing that antibody sequence representations capture substantial signal beyond CDR position. Mean-antigen (0.722) and shuffled-antigen (0.718) controls remained below full ParaEM (0.760). Their relatively strong performance indicates a major antibody-side contribution, while the matching antigen provides additional predictive information.

**Table 3:** Antibody-side and antigen-information controls on the PECAN dataset.^a^

Model/control	AUC-PR	AUC-ROC
CDR-only heuristic	0.326	0.845
Antibody-only ESM3	0.709	0.957
ParaEM + mean antigen	0.722	0.960
ParaEM + shuffled antigen	0.718±0.002	0.959±0.001
ParaEM	**0.760**	**0.968**

a Shuffled-antigen results are reported as mean ± SD over 30 random permutations. Detailed control settings are described in the Materials and methods section. Best values per metric are highlighted in bold.

### 3.5 External validation

To evaluate generalization beyond the original benchmarks, we constructed an external set of recently released SAbDab2 (Capel *et al*. 2026) antibody–antigen complexes with PDB release dates on or after 1 January 2025. Sequence redundancy relative to the PECAN training set was controlled using CD-HIT-2D thresholds of 95% for antibodies and 90% for antigens, retaining a complex only when both partners passed. After removing exact duplicate antibody–antigen sequence pairs and restricting complexes to at most two protein antigen chains, 247 complexes remained. The fixed PECAN-trained model was evaluated without retraining or parameter adjustment and achieved AUC-PR/AUC-ROC of 0.706/0.954; the processed set is available in the ParaEM GitHub repository.

### 3.6 Contact enrichment and anchor localization

To assess whether ParaEM’s learned score matrix *S* contains structure-related signal, we compared it with the residue–residue contact map derived from experimental antibody–antigen structures. Across the PECAN test set (n=152), *S* showed pair-level contact enrichment that covaried with paratope prediction quality. The high-AUC-PR group (top 20%) showed greater contact enrichment than the bottom 20%: ΔAUC-ROC=0.0387 (95% CI [0.0292, 0.0483]) and Δ(E[S∣C=1]−E[S∣C=0])=1.1028 ([0.7807, 1.4150]). Per-complex AUC-PR correlated with contact enrichment (Spearman ρ=0.679, Pearson r=0.710; both $p<10^{-20}$). Separately, macro Hit@1/3/5 values of 9.3%/21.5%/30.1% indicate partial interface-localization signal in the latent anchors despite the absence of antigen-side residue supervision, rather than explicit contact-prediction accuracy.

### 3.7 Exploratory antigen subgroup analysis

As an exploratory *post hoc* analysis, ParaEM showed lower mean AUC-PR for viral-surface antigens (0.676) than for membrane (0.776), extracellular (0.779), and unknown (0.786) antigens (Fig. 2; Table S2, available as supplementary data at *Bioinformatics* online). We therefore interpret this as a subgroup pattern observed within ParaEM. Supporting deterministic checks, robustness analyses, and topology diagnostics are provided in Text S2/Table S4, Table S3, and Text S4/Fig. S1, available as supplementary data at *Bioinformatics* online, respectively.

**Figure 2:**
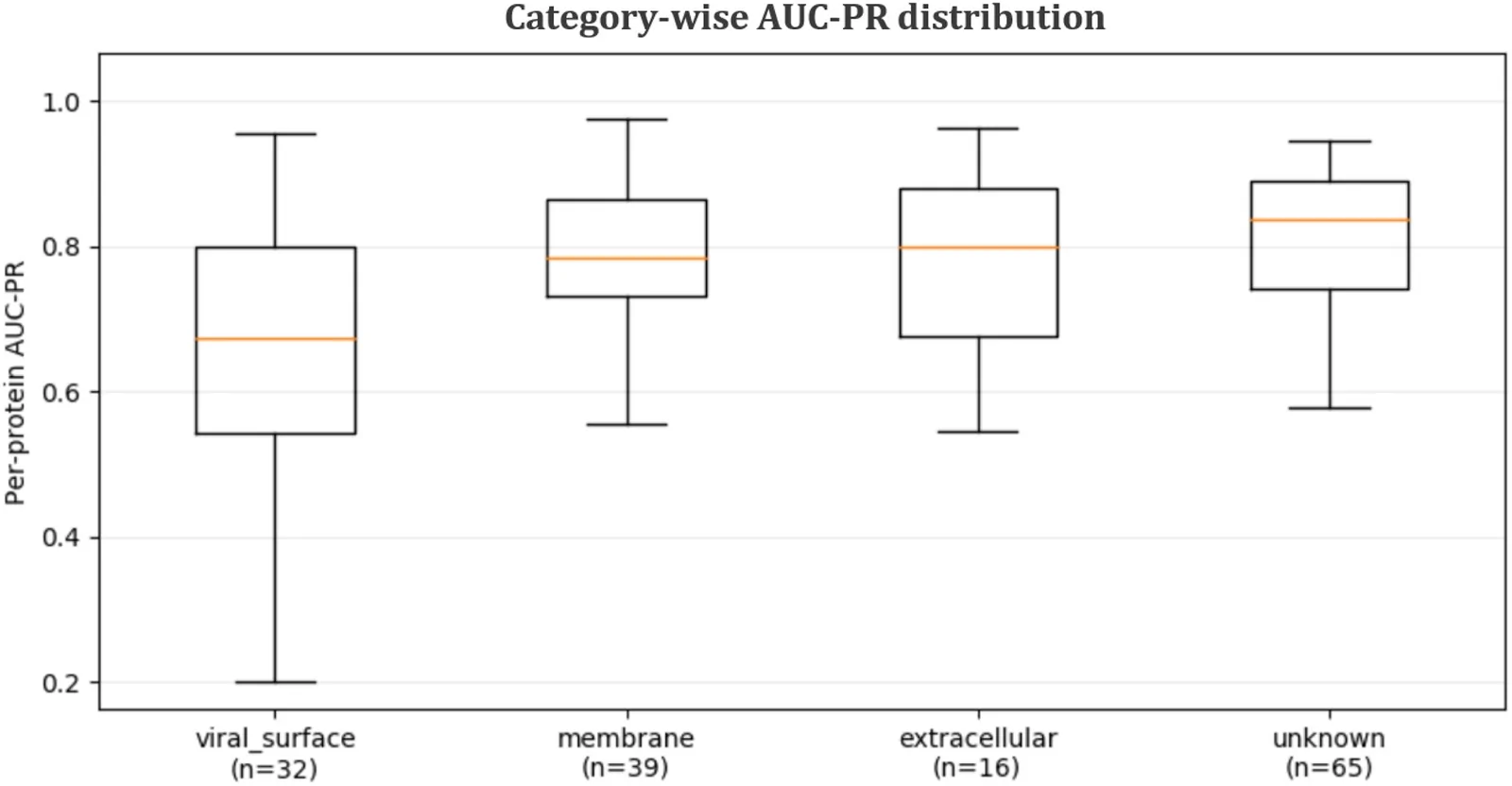
Distribution of per-complex AUC-PR across four coarse antigen localization categories (viral surface, membrane, extracellular, and unknown) derived from LLM-assisted annotation of UniProt metadata. Category sample sizes (*n*) are shown on the *x* axis.

## 4 Discussion

ParaEM is an antigen-aware sequence-based paratope predictor that combines pretrained protein embeddings with antibody–antigen compatibility scoring, a latent antigen-anchor formulation, generalized EM optimization, and CDR-informed embeddings. Across three established benchmarks, ParaEM achieved higher reported AUC-PR than existing sequence-based baselines and competitive performance relative to structure-based predictors while requiring only antibody and antigen sequences at inference time. The reduction in ParaSurf performance when experimental antibody structures were replaced with ColabFold-predicted structures further illustrates the practical dependence of structure-based prediction on the quality of structural inputs. Ablation studies showed consistent gains from generalized EM optimization, with additional improvements from CDR-informed embeddings. Antibody-side and antigen-information controls indicated that antibody sequence provides substantial predictive signal, while the matching antigen contributes additional predictive information. The learned compatibility scores showed aggregate contact enrichment, and the latent antigen anchors exhibited partial interface localization despite the absence of antigen-side residue supervision. The fixed PECAN-trained model also retained predictive performance on 247 recently released SAbDab2 complexes, achieving an AUC-PR of 0.706 and an AUC-ROC of 0.954 without retraining or parameter adjustment.

## 5 Conclusion

Overall, these results support ParaEM as a practical framework for incorporating matching-antigen context into sequence-based paratope prediction without requiring antibody–antigen structures at inference time. Future work will extend this framework toward joint paratope–epitope prediction by integrating sequence-based representations with three-dimensional structural information from both antibody and antigen partners.

## Supplementary Material

btag739_Supplementary_Data
