# Supplementary material for: ParaEM: antigen-aware sequence-based paratope prediction with expectation–maximization
Source: Bioinformatics. 2026 Oct 5;42(10):btag739. doi: 10.1093/bioinformatics/btag739 (PMC13676383; doi:10.1093/bioinformatics/btag739)
Supplement: Supplementary file 1 [file btag739_supplementary_data.pdf]

ParaEM: Antigen-aware sequence-based paratope prediction with Expectation-Maximization

Supplementary Material

Table of Contents

1.Supplementary Tables

Table S1. Robustness to train-test antigen sequence similarity

Table S2. Antigen localization subgroup performance of ParaEM and ParaSurf (pred)

Table S3. Robustness of the ParaEM viral-surface subgroup difference under resampling and permutation

Table S4. Agreement between LLM-assisted and deterministic UniProt category assignments

2. Supplementary Texts

Text S1. ColabFold-based sequence-only structure prediction for ParaSurf

Text S2. LLM-assisted UniProt annotation, deterministic cross-check, and residue-coordinate mapping

Text S3. Category-specific nonconformity quantiles as a descriptive calibration diagnostic

Text S4. Argmax-based topology diagnostic for the illustrative Figure S1 case study

3. Supplementary Figures

Figure S1. Illustrative topology-oriented case study of the 5LBS viral-surface antigen

1. Supplementary Tables

Table S1. Robustness to train-test antigen sequence similarity

For each benchmark, CD-HIT-2D was used to compare antigen sequences from the provided training and test splits at sequence-identity cutoffs of 70%, 60%, 50%, and 40%. At each cutoff, test complexes whose antigen was identified as similar to any training antigen at or above the specified threshold were excluded. The original training data and trained ParaEM models were left unchanged; no retraining was performed, and performance was reevaluated only on the retained test subset.

Dataset	Sequence-identity cutoff	n	AUC-PR	AUC-ROC
PECAN	Original	152	0.760	0.968
PECAN	<70%	100	0.769	0.968
PECAN	<60%	99	0.770	0.968
PECAN	<50%	97	0.771	0.968
PECAN	<40%	90	0.773	0.969
Paragraph Expanded	Original	217	0.786	0.971
Paragraph Expanded	<70%	98	0.789	0.969
Paragraph Expanded	<60%	94	0.793	0.969
Paragraph Expanded	<50%	91	0.794	0.969
Paragraph Expanded	<40%	90	0.798	0.970
MIPE	Original	63	0.757	0.968
MIPE	<70%	59	0.754	0.965
MIPE	<60%	58	0.751	0.964
MIPE	<50%	55	0.753	0.964
MIPE	<40%	53	0.752	0.964

Interpretation. The subset labels <70%, <60%, <50%, and <40% denote test complexes retained after excluding antigens that met or exceeded the corresponding train-test sequence-identity threshold. Performance remained broadly comparable as increasingly similar test antigens were removed. At the 40% cutoff, 90 PECAN, 90 Paragraph Expanded, and 53 MIPE test complexes were retained, with no material loss of predictive performance. These results indicate that residual train-test antigen sequence similarity is unlikely to be a major driver of the reported benchmark performance.

Table S2. Antigen localization subgroup performance of ParaEM and ParaSurf (pred)

Category	n	ParaEM Mean AUC-PR	ParaSurf (pred)Mean AUC-PR
Viral_surface	32	0.676	0.675
Membrane	39	0.776	0.784
Extracellular	16	0.779	0.800
Unknown	65	0.786	0.820

Per-complex AUC-PR across the four protein-level antigen localization categories used for exploratory post-hoc subgroup analysis on the PECAN test set. The same category assignments were applied to ParaEM and ParaSurf (pred), where ParaSurf (pred) denotes ParaSurf evaluated using ColabFold-predicted antibody structures. The lower viral-surface performance observed for ParaEM was not reproduced by ParaSurf (pred), supporting our interpretation of this result as a subgroup pattern observed within ParaEM rather than evidence that viral-surface antigens are uniformly more difficult across predictors.

Table S3. Robustness of the ParaEM viral-surface subgroup difference under resampling and permutation

Statistic	Viral_surface(n=32) vs Others (n=120)
Δmean AUC-PR	-0.106
Bootstrap 95% CI	(-0.190, -0.043)
Cliff’s delta	-0.361
Permutation test p	0.0007

To assess the robustness of the subgroup difference observed within ParaEM, we compared per-complex AUC-PR for viral-surface antigens with all remaining categories pooled. A nonparametric bootstrap resampled complexes with replacement within each group to estimate the 95% confidence interval for the mean difference. Cliff's delta was used as a distributional effect size, and a label-permutation test evaluated the observed mean difference under a two-group null distribution. These analyses quantify the stability of the subgroup difference observed within ParaEM and are not intended to establish that viral-surface antigens are intrinsically more difficult across prediction methods.

Table S4. Agreement between LLM-assisted and deterministic UniProt category assignments

LLM-assisted assignment	Deterministic: viral surface	Deterministic: membrane	Deterministic: extracellular	Deterministic: unknown	Row total
Viral surface	29	3	0	0	32
Membrane	5	34	0	0	39
Extracellular	0	5	11	0	16
Unknown	1	9	6	2	18
Column total	35	51	17	2	105

Agreement matrix for the 105 PECAN test antigens with a qualifying BLASTP/UniProt match. Rows indicate LLM-assisted categories and columns indicate categories assigned by the deterministic UniProt rules described in Text S2. Overall agreement was 76/105 (72.4%). The 47 antigens without a qualifying BLASTP/UniProt match were retained as unknown by design and are excluded from this agreement denominator. Because both procedures use the same underlying UniProt records, this analysis is an LLM-independent deterministic consistency cross-check of the category-assignment procedure rather than external biological ground-truth validation.

2. Supplementary Texts

Text S1. ColabFold-based sequence-only structure prediction for ParaSurf

To examine ParaSurf performance when experimental antibody structures are unavailable, antibody structures were generated from sequence using ColabFold. For each test antibody in PECAN, Paragraph Expanded, and MIPE, a separate structure prediction was performed using the VH and VL variable-region sequences as query inputs. VH and VL were supplied as separate chains and jointly modeled using AlphaFold2-multimer v3. The top-ranked predicted antibody structure was used as input for ParaSurf (pred), replacing antibody coordinates extracted from the experimentally determined antibody-antigen complex.

Independence from bound-complex information. The antibody and antigen were never provided jointly to ColabFold as a bound antibody-antigen complex. Where antigen structures were generated for auxiliary analyses, they were predicted separately using the antigen sequence(s) as query inputs; for multichain antigens, only antigen chains were supplied together. Template search was not enabled, and no experimental structures from the benchmark antibody-antigen complexes were supplied as templates. No experimental complex coordinates, interface geometry, residue-contact labels, docking pose, or other benchmark-specific bound-complex information was provided during structure prediction. Experimentally determined complexes were used only to define benchmark labels and for downstream evaluation.

- **Command (antibody multimer prediction).**
  colabfold_batch antibody_multimer.fasta result --msa-mode mmseqs2_uniref_env --model-type alphafold2_multimer_v3 --num-models 5 --num-recycle 3 --stop-at-score 85
- **MSA retrieval (--msa-mode mmseqs2_uniref_env).**
  ColabFold's MMseqs2-based pipeline was used to construct MSAs from UniRef and environmental sequence resources.
- **Model configuration (--model-type alphafold2_multimer_v3).**
  The AlphaFold2-multimer v3 model was used for the paired VH/VL input and for multichain antigen inputs when applicable.
- **Ensembling (--num-models 5).**
  For each antibody, five model parameter sets were evaluated (the standard AF2 model ensemble), producing up to five candidate structures.
- **Recycling (--num-recycle 3).**
  We used three recycling iterations per model to refine the predicted structure.
- **Early stopping (--stop-at-score 85).**
  We enabled ColabFold’s early-stopping criterion to terminate recycling when model confidence exceeded the specified threshold, reducing compute without materially affecting high-confidence cases.

Text S2. LLM-assisted UniProt annotation, deterministic cross-check, and residue-coordinate mapping

Protein-level annotation for subgroup analysis. For the exploratory post-hoc antigen analysis, each PECAN test antigen was linked to its top-ranked UniProt homolog using BLASTP. Retrieved BLASTP and UniProt metadata were provided to an LLM-assisted annotation procedure that organized the evidence into predefined protein-level descriptors, from which each antigen was assigned to one of four coarse localization categories: viral surface, membrane, extracellular, or unknown. Antigens without a qualifying BLASTP/UniProt match were conservatively retained as unknown. The structured outputs used for category assignment are provided in the ParaEM GitHub repository. These annotations were used only for downstream subgroup analysis and did not affect ParaEM training or inference.

Deterministic UniProt consistency cross-check. To cross-check the LLM-assisted categories without using an LLM, we parsed the corresponding UniProt records using deterministic rules based on the DE, OS, OC, CC SUBCELLULAR LOCATION, GO cellular-component, KW, FT TRANSMEM, and FT SIGNAL fields. Categories were assigned with the precedence viral surface > membrane > extracellular > unknown. A record was classified as viral surface only when viral taxonomy was accompanied by a predefined surface-associated protein description (for example, spike/surface/envelope glycoprotein, hemagglutinin, neuraminidase, gp120/gp160/gp41, fusion or attachment glycoprotein, or coat/capsid protein); viral taxonomy alone was insufficient. A record was classified as membrane when an FT TRANSMEM feature or explicit membrane localization annotation was present. Extracellular required explicit secreted/extracellular localization evidence; a signal-peptide annotation alone was not sufficient. Records not meeting these criteria were assigned unknown.

Agreement with the deterministic rules. Of the 152 PECAN test antigens, 47 lacked a qualifying BLASTP/UniProt match and were retained as unknown by design. Among the remaining 105 antigens with UniProt records, 76/105 assignments (72.4%) agreed with the deterministic classification, corresponding to Cohen's $\kappa = 0.600$ (Table S4). Disagreements occurred predominantly at boundaries involving the membrane category, consistent with overlapping or context-dependent protein-level localization annotations. Because both procedures draw on the same UniProt records, this analysis is interpreted as an LLM-independent consistency cross-check of the category-assignment procedure rather than external biological validation.

Residue-level topology annotation for the illustrative case study. Separately from the protein-level subgroup annotation, an independent residue-level procedure was used to obtain topology segments from UniProt feature annotations for the illustrative

case study in Figure S1. Segment records contained start and end positions, a topology label, confidence, and supporting evidence. The topology vocabulary included transmembrane, extracellular, luminal, cytosolic, signal peptide, and unknown. These residue-level annotations were not used to define the four protein-level antigen categories.

Mapping UniProt feature coordinates to the PDB-derived antigen sequence. UniProt residue numbers were not assumed to correspond directly to residue indices in the PDB-derived antigen sequence. An explicit residue mapping was constructed from the UniProt sequence (BLASTP subject) to the PDB-derived antigen sequence (BLASTP query) using the gapped qseq/sseq alignment returned by BLASTP. Query and subject indices were advanced only at non-gap alignment positions, establishing residue correspondences for aligned positions. UniProt feature-table annotations were then projected from subject coordinates to corresponding query coordinates. Residues outside the aligned region or without a query correspondence because of alignment gaps were not directly projected. Consequently, the topology segments in Figure S1 are expressed in the coordinate system of the actual PDB-derived antigen sequence.

Text S3. Category-specific nonconformity quantiles as a descriptive calibration diagnostic

For each antigen category

$g \in \{viral\_surface, membrane, extracellular, unknown\}$, antibody-residue predictions in the validation split whose paired antigen belonged to category g were collected.

For residue i with ground-truth label $y_i \in \{0, 1\}$, and predicted paratope probability $\hat{p}_i$, the nonconformity score is defined as the probability assigned to the incorrect label:

$$s_i = \begin{cases} 1 - \widehat{p_i}, & y_i = 1 \\ \widehat{p_i}, & y_i = 0 \end{cases}$$

Let n_g denote the number of calibration residues in category g, and let

$$s_1^g \leq \cdots \leq s_{n_g}^g$$

denote the sorted nonconformity scores. We report the finite-sample $(1 - \alpha)$ nonconformity quantile $\hat{q}_g$ with $\alpha = 0.1$ as a category-specific descriptive calibration statistic.

$$\hat{q}_g = s_k^g, \qquad k = \left[(n_g + 1)(1 - \alpha) \right].$$

Larger $\hat{q}_g$ values indicate larger empirical nonconformity within that validation category. The resulting $\hat{q}_g$ values were 0.356, 0.274, 0.231, and 0.278 for the viral-surface, membrane, extracellular, and unknown categories, respectively.

Because empirical test-set coverage, average prediction-set size, and singleton/empty-set frequencies are not reported, these quantities are not interpreted as establishing a formal conformal coverage guarantee and are not used during ParaEM training or inference.

Text S4. Argmax-based topology diagnostic for the illustrative Figure S1 case study

To examine how ParaEM's learned antigen-anchor weight relate to antigen topology annotations, we used an argmax-based diagnostic. For each complex, let $\pi_{i,j}$ denote the anchor weight over antigen positions for antibody residue i, with $\sum_j \pi_{i,j} = 1$. We define the peak anchor position for residue i as

$$j^*(i) = argmax_j \pi_{i,j} ,$$

breaking ties by selecting the smallest index.

Given an antigen segment mask $m^{seg} \in \{0, 1\}^{L_{Ag}}$, we define

$$frac\_argmax\_seg = \frac{1}{L_{Ab}} \sum_{i=1}^{L_{Ab}} \mathbb{I}[m^{seg}_{j^*(i)} = 1].$$

Given a binary antigen-segment mask over query-coordinate antigen positions, frac_argmax_seg is defined as the fraction of antibody residues whose peak anchor position falls within that segment class. Figure S1 reports frac_argmax_surface for surface-facing extracellular/luminal segments and frac_argmax_TM for transmembrane segments. The residue-level topology masks are defined in PDB-antigen query coordinates after projecting UniProt feature annotations through the gapped BLASTP qseq/sseq mapping described in Text S2.

This analysis is presented only as an illustrative topology-oriented diagnostic for one low-performing viral-surface case and is independent of the protein-level category assignment used for subgroup stratification.

3. Supplementary Figures

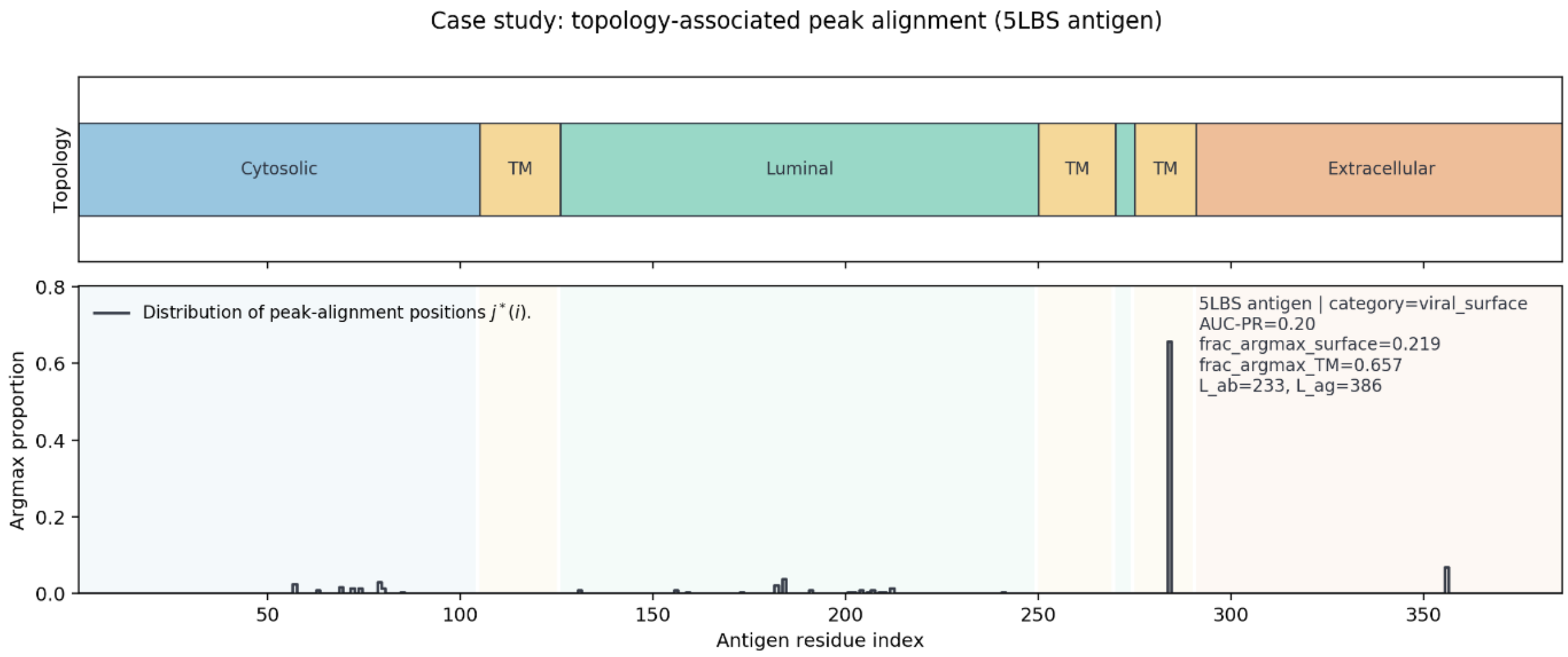

Figure S1. Illustrative topology-oriented case study of the 5LBS viral-surface antigen

The 5LBS antigen is shown as an illustrative low-performing viral-surface example. **Top:** residue-level topology annotation of the antigen derived from UniProt feature annotations, showing cytosolic, transmembrane, luminal, and extracellular segments after projection from UniProt subject coordinates to the PDB-derived antigen query coordinates using the gapped BLASTP qseq/sseq alignment described in Text S2. **Bottom:** distribution of peak antigen-anchor positions across antibody residues, where the peak position for residue i is the antigen position with the largest row-wise antigen-anchor weight. The inset reports frac_argmax_surface and frac_argmax_TM, defined as the fraction of antibody residues whose peak anchor falls in surface-facing (extracellular/luminal) or transmembrane antigen segments, respectively. In this example, frac_argmax_TM = 0.657 and frac_argmax_surface = 0.219. This figure is provided as an illustrative diagnostic and is not used to define the protein-level antigen categories or to establish a general causal mechanism for the viral-surface performance difference.
